# Antimicrobial resistance in India

**DOI:** 10.1186/s40545-017-0118-6

**Published:** 2017-09-05

**Authors:** Isha Patel, Rabia Hussain, Anam Khan, Akram Ahmad, Muhammad Umair Khan, Mohamed Azmi Ahmed Hassalai

**Affiliations:** 1 Department of Pharmacy Practice, Administration and Research, Marshall University School of Pharmacy (MUSOP), Marshall University, Huntington, WV 25755 USA; 2 Discipline of Social and Administrative Pharmacy, School of Pharmaceutical Sciences, Universiti Sains Malaysia, Penang, Malaysia; 3 Tech Observer, 1391/34A, Nangal Raya, Janakpuri, New Delhi, 110046 India; 4 Department of Clinical Pharmacy, Faculty of Pharmaceutical Sciences, UCSI University, Kuala Lumpur, Malaysia; 5 Faculty of Pharmacy, The University of Sydney, Sydney, NSW Australia

In April 2017, Indian Council of Medical Research advised 20 tertiary care hospitals in South India to put a strict control on the use carbapenems and polymyxins and labelled them as high need or end use antibiotics [1]. This was in the context that these antibiotics were considered the last treatment options for the infections resistant to all other available antibiotics [2, 3]. This was coming from a study which was conducted in the Intensive Care Units of 20 tertiary care hospitals and it was observed that 5-7% critically ill patients are resistant to antibiotics [1]. Notably, antibiotic resistance and overuse of antibiotics, especially in ICU contribute towards life threatening condition such as sepsis and even death. It is reported that every 1 in 7 catheter and surgery related infections can be caused by antibiotic resistant bacteria including Carbapenem-resistant Enterobacteriaceae [4].

Overall, the emergence of infections by antibiotic resistant Gram positive and Gram negative microbes is on a great rise, taking a note on *Escherichia coli,* which is an increasing global concern for the resistance to antibiotics and in India the resistance is found to be more than 80% for these classes of antibiotics. When 66% surgical infections are caused by Gram negative bacilli, a study reported in 2013 that 13% of the *E.Coli* strain showed resistance to the last line of therapy antibiotics, such as Carbapenems. Likewise, an increased emergence of methicillin-resistant *Staphylococcus aureus* (MRSA)*,* causing 54.8% surgical infections, was documented in India, and a steep rise in resistance was found from an isolation percentage of 29% in 2009 to 47% in 2014 from private labs [5].

To control the antimicrobial resistance globally, comprehensive policies on antibiotics use are needed while different countries are at different stages of development of these policies. This could include bringing systematic interventions to educate healthcare professionals about prescribing antibiotics, developing infections control guidelines and keeping a control on the marketing and sales of the antibiotics. Similarly, many hospitals in India have established policies to minimize the surgical infections as patients are directly exposed to the serious antibiotic resistant microbes in health care facilities [6]. But, in spite of the efforts being made, the time is running out and perhaps actions are urgently needed to track the resistance mechanisms of infectious organisms by rationalizing the use of antibiotics through the formulation of antibiotic policies and antibiotic stewardship programmes in the country.
